# Supplementary material for: Comparative Genomics and Transcriptomics Analyses Reveal Divergent Plant Biomass-Degrading Strategies in Fungi
Source: J Fungi (Basel). 2023 Aug 18;9(8):860. doi: 10.3390/jof9080860 (PMC10455118; doi:10.3390/jof9080860)
Supplement: Supplementary file 1 [file jof-09-00860-s001.zip › Figure S3.pdf]

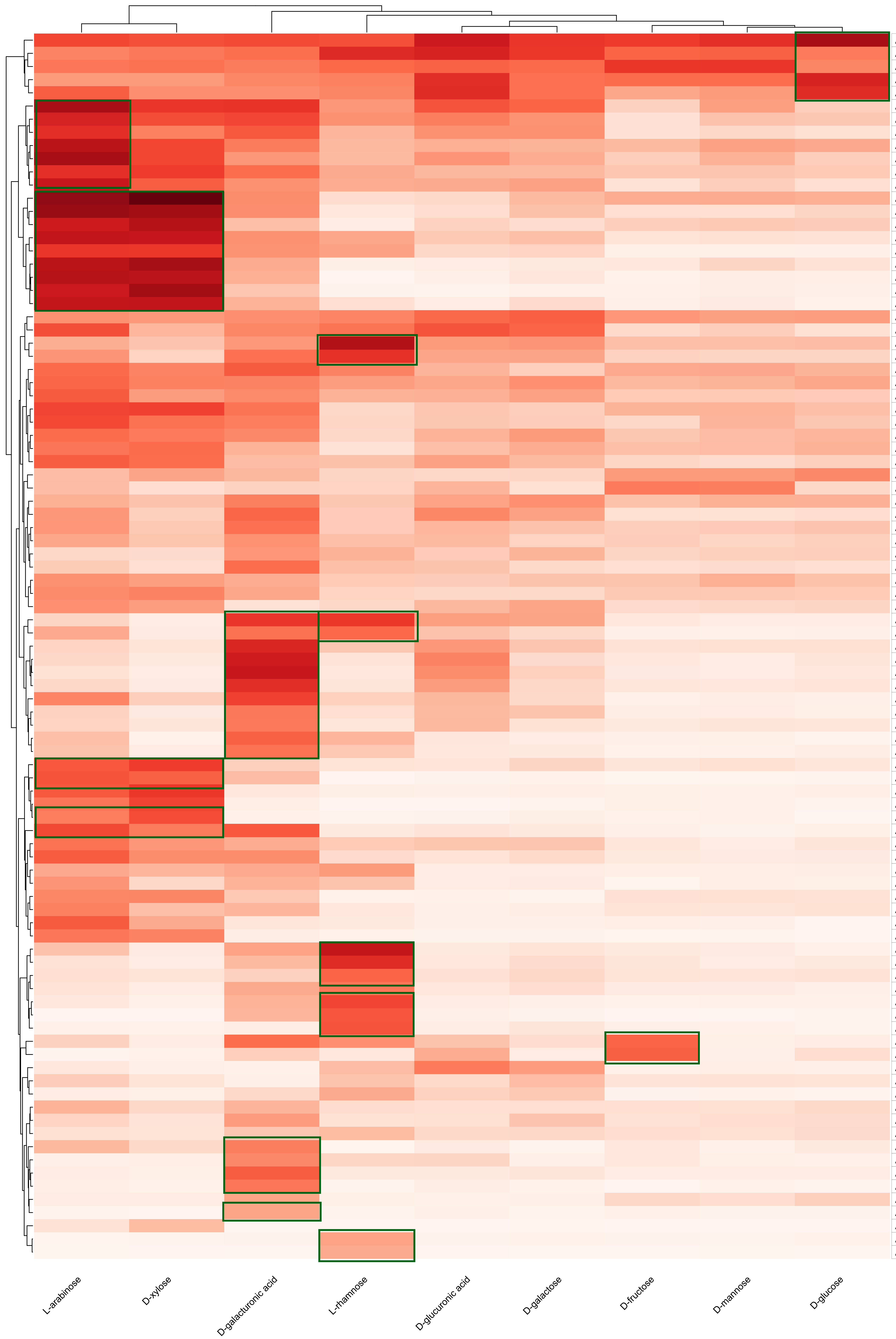

GeneID	CAZy family	Activity	Substrate
Aspni_NRRL3_1|8300	GH15	GLA	Starch
Aspni_NRRL3_1|5407	GH43	ABN	Pectin
Aspni_NRRL3_1|8517	GH1	BGL	Cellulose
Aspni_NRRL3_1|7700	GH31	AGD	Starch
Aspni_NRRL3_1|9875	GH13_1	AMY	Starch
Aspni_NRRL3_1|3768	GH54	ABF	Xylan/Pectin/Xyloglucan
Aspni_NRRL3_1|2630	GH35	LAC	Pectin/Xyloglucan
Aspni_NRRL3_1|10865	GH51	ABF	Xylan/Pectin/Xyloglucan
Aspni_NRRL3_1|8701	GH5_16	ENDO-BETA-1,6-GALACTANASE	Pectin
Aspni_NRRL3_1|1606	GH51	ABF	Xylan/Pectin/Xyloglucan
Aspni_NRRL3_1|1918	GH12	XEG	Xyloglucan
Aspni_NRRL3_1|743	GH27	AGL	Mannan
Aspni_NRRL3_1|1648	GH11	XLN	Xylan
Aspni_NRRL3_1|8707	GH62	AXH	Xylan
Aspni_NRRL3_1|819	GH12	EGL	Cellulose
Aspni_NRRL3_1|2451	GH3	BXL	Xylan
Aspni_NRRL3_1|1069	GH67	AGU	Xylan
Aspni_NRRL3_1|3928	GH11	XLN	Xylan
Aspni_NRRL3_1|8708	GH10	XLN	Xylan
Aspni_NRRL3_1|7	FAE	\	Pectin/Xylan
Aspni_NRRL3_1|3339	CE1	AXE	Xylan
Aspni_NRRL3_1|9890	GH43	ABF/BXL	Xylan
Aspni_NRRL3_1|92	GH43	ABN	Pectin
Aspni_NRRL3_1|2162	GH78	RHA	Pectin
Aspni_NRRL3_1|7501	CE12	RGAE	Pectin
Aspni_NRRL3_1|7470	CE8	PME	Pectin
Aspni_NRRL3_1|6791	GH5_5	EGL	Cellulose
Aspni_NRRL3_1|2023	GH31	AXL	Xyloglucan
Aspni_NRRL3_1|268	GH31	AXL	Xyloglucan
Aspni_NRRL3_1|6387	GH51	ABF	Xylan/Pectin/Xyloglucan
Aspni_NRRL3_1|814	AA9	LPMO	Cellulose
Aspni_NRRL3_1|3929	AA9	LPMO	Cellulose
Aspni_NRRL3_1|9644	GH3	BGL	Cellulose
Aspni_NRRL3_1|6316	AA3_1	CDH	Cellulose
Aspni_NRRL3_1|6436	GH3	BGL	Cellulose
Aspni_NRRL3_1|2656	AA16	LPMO	Cellulose
Aspni_NRRL3_1|2479	GH35	LAC	Pectin/Xyloglucan
Aspni_NRRL3_1|2571	GH28	PGA	Pectin
Aspni_NRRL3_1|10643	GH53	GAL	Pectin
Aspni_NRRL3_1|7411	CE1	AXE	Xylan
Aspni_NRRL3_1|965	PL1_4	PEL	Pectin
Aspni_NRRL3_1|9612	GH2	MND	Mannan
Aspni_NRRL3_1|4953	GH7	CBH	Cellulose
Aspni_NRRL3_1|3961	AA9	LPMO	Cellulose
Aspni_NRRL3_1|169	CE12	RGAE	Pectin
Aspni_NRRL3_1|11738	GH35	LAC	Pectin/Xyloglucan
Aspni_NRRL3_1|6053	CE16	HAE	Xylan/Mannan/Xyloglucan
Aspni_NRRL3_1|3144	GH28	PGX	Pectin
Aspni_NRRL3_1|8281	GH28	PGX	Pectin
Aspni_NRRL3_1|5252	CE8	PME	Pectin
Aspni_NRRL3_1|4916	CE16	HAE	Xylan/Mannan/Xyloglucan
Aspni_NRRL3_1|6244	GH43	ABF/BXL	Xylan
Aspni_NRRL3_1|5260	GH28	PGA	Pectin
Aspni_NRRL3_1|10559	GH28	RGX	Pectin
Aspni_NRRL3_1|10558	GH78	RHA	Pectin
Aspni_NRRL3_1|10881	GH43	ABF/BXL	Xylan
Aspni_NRRL3_1|7382	GH95	AFC	Xyloglucan
Aspni_NRRL3_1|10870	GH6	CBH	Cellulose
Aspni_NRRL3_1|9019	GH6	CBH	Cellulose
Aspni_NRRL3_1|8993	CE1	AXE	Xylan
Aspni_NRRL3_1|6379	CE16	AXE	Xylan
Aspni_NRRL3_1|9976	GH1	BGL	Cellulose
Aspni_NRRL3_1|6419	GH3	BGL/BXL	Cellulose/Xylan
Aspni_NRRL3_1|1071	GH35	LAC	Pectin/Xyloglucan
Aspni_NRRL3_1|1737	GH27	AGL	Mannan
Aspni_NRRL3_1|1787	GH74	XEG	Xyloglucan
Aspni_NRRL3_1|7469	GH28	XGH	Pectin
Aspni_NRRL3_1|3383	AA9	LPMO	Cellulose
Aspni_NRRL3_1|4917	GH5_5	EGL	Cellulose
Aspni_NRRL3_1|10115	PL4_3	RGL	Pectin
Aspni_NRRL3_1|839	GH105	URGH	Pectin
Aspni_NRRL3_1|7520	GH78	RHA	Pectin
Aspni_NRRL3_1|2827	GH11	XLN	Xylan
Aspni_NRRL3_1|2832	GH28	RGX	Pectin
Aspni_NRRL3_1|8631	GH28	RGX	Pectin
Aspni_NRRL3_1|3279	GH78	RHA	Pectin
Aspni_NRRL3_1|11752	GH32	INV	Inulin
Aspni_NRRL3_1|3087	GH32	INX	Inulin
Aspni_NRRL3_1|3855	GH43	ABN	Pectin
Aspni_NRRL3_1|6359	PL1_7	PLY	Pectin
Aspni_NRRL3_1|7975	GH1	BGL	Cellulose
Aspni_NRRL3_1|263	GH28	PGA	Pectin
Aspni_NRRL3_1|11105	GH131	EGL	Cellulose
Aspni_NRRL3_1|2931	FAE	\	Pectin/Xylan
Aspni_NRRL3_1|9810	GH28	PGX	Pectin
Aspni_NRRL3_1|4000	GH28	PGA	Pectin
Aspni_NRRL3_1|2835	GH28	PGA	Pectin
Aspni_NRRL3_1|8805	GH28	PGA	Pectin
Aspni_NRRL3_1|9001	GH13_1	AMY	Starch
Aspni_NRRL3_1|8325	CE8	PME	Pectin
Aspni_NRRL3_1|9623	GH28	RHG	Pectin
Aspni_NRRL3_1|6304	GH78	RHA	Pectin
Aspni_NRRL3_1|4245	GH78	RHA	Pectin

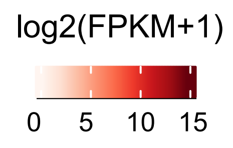

A. niger

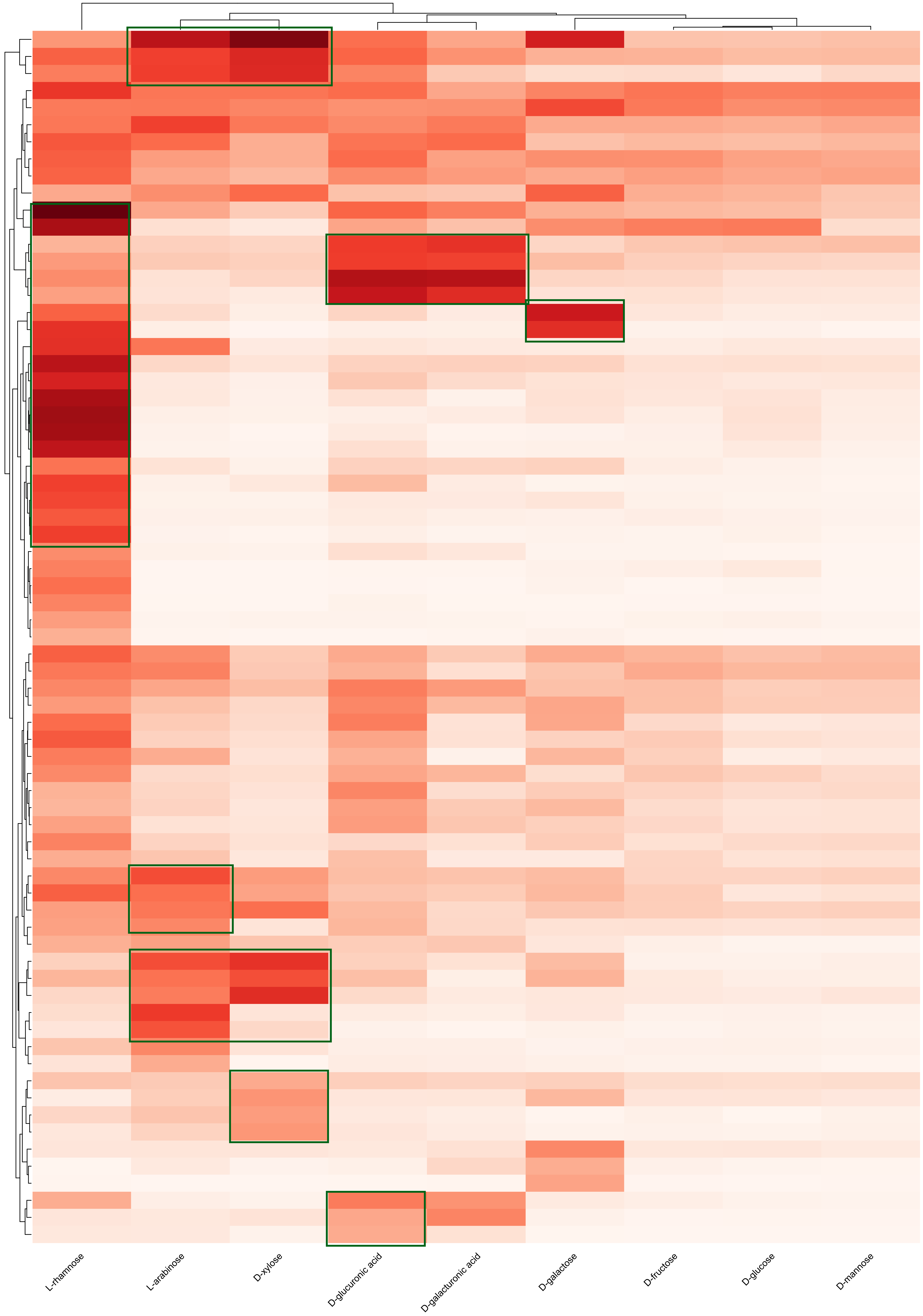

GeneID	CAZy family	Activity	Substrate
Aspnid1|8110	GH3	BXL	Xylan
Aspnid1|7119	GH43	ABF/BXL	Xylan
Aspnid1|1280	GH43	ABF/BXL	Xylan
Aspnid1|1998	GH3	BGL	Cellulose
Aspnid1|9090	GH43	ABN	Pectin
Aspnid1|5409	GH51	ABF	Xylan/Pectin/Xyloglucan
Aspnid1|3324	GH31	AXL	Xyloglucan
Aspnid1|7563	GH43	ABF/BXL	Xylan
Aspnid1|5735	GH35	LAC	Pectin/Xyloglucan
Aspnid1|1435	GH43	ABN	Pectin
Aspnid1|8918	GH105	URGH	Pectin
Aspnid1|6930	GH31	AGD	Starch
Aspnid1|267	CE8	PME	Pectin
Aspnid1|5232	GH105	URGH	Pectin
Aspnid1|7002	GH28	PGX	Pectin
Aspnid1|1049	GH28	PGX	Pectin
Aspnid1|8912	GH2	LAC	Pectin/Xyloglucan
Aspnid1|2120	GH88	UGH	Pectin
Aspnid1|3654	GH43	ABF	Pectin/Xyloglucan
Aspnid1|3832	PL4_3	RGL	Pectin
Aspnid1|5016	GH78	RHA	Pectin
Aspnid1|7934	GH28	RGX	Pectin
Aspnid1|3024	GH78	RHA	Pectin
Aspnid1|7933	GH78	RHA	Pectin
Aspnid1|1957	PL4_3	RGL	Pectin
Aspnid1|8705	GH13_1	AMY	Starch
Aspnid1|7478	CE16	AXE	Xylan
Aspnid1|9325	CE12	RGAE	Pectin
Aspnid1|8467	GH1	BGL	Cellulose
Aspnid1|9766	GH78	RHA	Pectin
Aspnid1|9721	GH88	UGH	Pectin
Aspnid1|10445	AA13	LPMO	Starch
Aspnid1|2169	GH3	BGL	Cellulose
Aspnid1|2748	GH3	BGL	Cellulose
Aspnid1|4176	AA13	LPMO	Starch
Aspnid1|8691	GH13_1	AMY	Starch
Aspnid1|8232	GH2	LAC	Pectin/Xyloglucan
Aspnid1|7769	GH93	ABX	Pectin
Aspnid1|7945	GH3	BGL/BXL	Cellulose/Xylan
Aspnid1|6988	GH15	GLA	Starch
Aspnid1|7454	FAE	\	Pectin/Xylan
Aspnid1|9332	GH3	BGL	Cellulose
Aspnid1|1583	GH36	AGL	Mannan
Aspnid1|2674	GH13_40	AGD	Starch
Aspnid1|6028	GH3	BGL	Cellulose
Aspnid1|6030	GH78	RHA	Pectin
Aspnid1|5590	GH2	MND	Mannan
Aspnid1|8306	CE12	RGAE	Pectin
Aspnid1|9685	GH36	AGL	Mannan
Aspnid1|10613	GH78	RHA	Pectin
Aspnid1|1595	GH95	AFC	Xyloglucan
Aspnid1|6505	GH31	AXL	Xyloglucan
Aspnid1|5977	GH35	LAC	Pectin/Xyloglucan
Aspnid1|9020	GH1	BGL	Cellulose
Aspnid1|1330	GH62	AXH	Xylan
Aspnid1|5129	GH67	AGU	Xylan
Aspnid1|7505	GH10	XLN	Xylan
Aspnid1|2882	GH43	ABF/BXL	Xylan
Aspnid1|2883	GH43	ABF/BXL	Xylan
Aspnid1|3023	GH27	AGL	Mannan
Aspnid1|7231	GH54	ABF	Xylan/Pectin/Xyloglucan
Aspnid1|4188	CE1	AXE	Xylan
Aspnid1|5214	GH11	XLN	Xylan
Aspnid1|2743	GH10	XLN	Xylan
Aspnid1|2560	GH11	XLN	Xylan
Aspnid1|2143	GH105	URGH	Pectin
Aspnid1|3436	GH131	EGL	Cellulose
Aspnid1|8490	GH5_16	ENDO−BETA−1,6−GALACTANASE	Pectin
Aspnid1|3708	GH105	URGH	Pectin
Aspnid1|4821	GH43	ABF/BXL	Xylan
Aspnid1|9694	GH3	BXL	Xylan

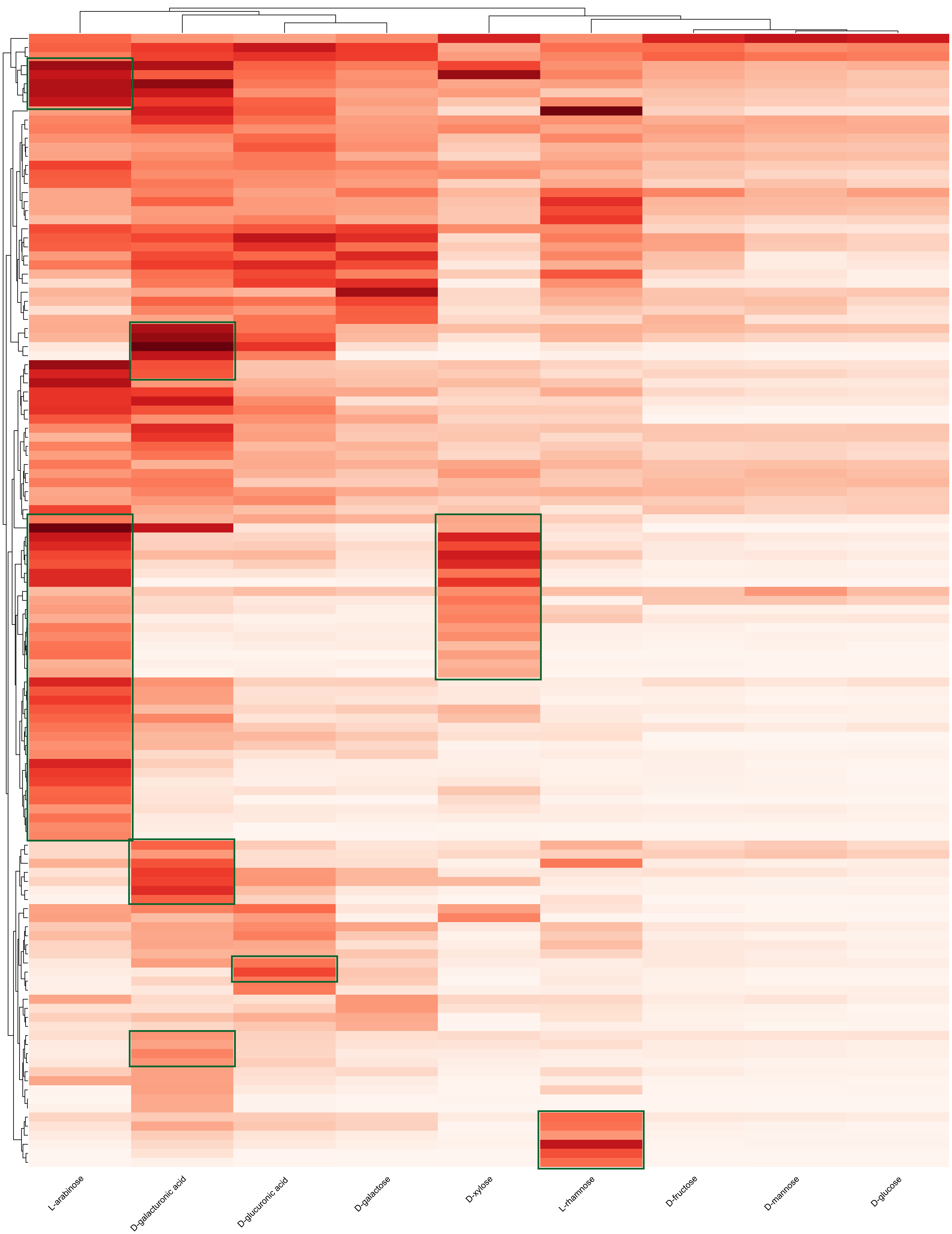

GeneID	CAZy family	Activity	Substrate
Pensub1|10072	GH13_1	AMY	Starch
Pensub1|12027	GH3	BGL	Cellulose
Pensub1|5932	GH43	ABN	Pectin
Pensub1|2281	GH51	ABF	Xylan/Pectin/Xyloglucan
Pensub1|13865	GH43	ABF/BXL	Xylan
Pensub1|8128	GH93	ABX	Pectin
Pensub1|13297	GH51	ABF	Xylan/Pectin/Xyloglucan
Pensub1|6338	GH35	LAC	Pectin/Xyloglucan
Pensub1|6624	GH105	URGH	Pectin
Pensub1|5035	GH28	PGA	Pectin
Pensub1|6307	AA16	LPMO	Cellulose
Pensub1|10447	GH27	AGL	Mannan
Pensub1|10078	GH36	AGL	Mannan
Pensub1|7144	GH3	BGL	Cellulose
Pensub1|11443	GH35	LAC	Pectin/Xyloglucan
Pensub1|5870	GH11	XLN	Xylan
Pensub1|4688	GH31	AXL	Xyloglucan
Pensub1|7928	GH15	GLA	Starch
Pensub1|9802	PL4_3	RGL	Pectin
Pensub1|13664	GH115	AGU	Xylan
Pensub1|11222	GH78	RHA	Pectin
Pensub1|2053	GH27	AGL	Mannan
Pensub1|848	GH27	AGL	Mannan
Pensub1|7307	GH32	INX	Inulin
Pensub1|8031	GH15	GLA	Starch
Pensub1|11945	GH2	LAC	Pectin/Xyloglucan
Pensub1|2371	GH43	ABN	Pectin
Pensub1|532	CE5	AXE	Xylan
Pensub1|11416	GH2	LAC	Pectin/Xyloglucan
Pensub1|362	GH13_40	AGD	Starch
Pensub1|361	GH13_40	AGD	Starch
Pensub1|10931	GH32	INV	Inulin
Pensub1|759	GH28	PGX	Pectin
Pensub1|2263	CE8	PME	Pectin
Pensub1|1655	GH28	PGX	Pectin
Pensub1|10256	GH28	PGA	Pectin
Pensub1|11001	GH43	ABF	Pectin/Xyloglucan
Pensub1|4395	GH27	AGL	Mannan
Pensub1|3399	GH62	AXH	Xylan
Pensub1|8890	GH1	BGL	Cellulose
Pensub1|4796	GH3	BGL/BXL	Cellulose/Xylan
Pensub1|11908	GH95	AFC	Xyloglucan
Pensub1|3477	GH27	AGL	Mannan
Pensub1|9719	GH35	LAC	Pectin/Xyloglucan
Pensub1|14178	GH28	PGA	Pectin
Pensub1|12847	GH2	MND	Mannan
Pensub1|12201	GH53	GAL	Pectin
Pensub1|6027	GH62	AXH	Xylan
Pensub1|11114	GH5_5	EGL	Cellulose
Pensub1|6250	GH1	BGL	Cellulose
Pensub1|6581	AA9	LPMO	Cellulose
Pensub1|9639	GH3	BGL	Cellulose
Pensub1|2294	GH7	CBH	Cellulose
Pensub1|9765	GH29	AFC	Xyloglucan
Pensub1|1940	GH54	ABF	Xylan/Pectin/Xyloglucan
Pensub1|3510	GH43	ABF/BXL	Xylan
Pensub1|11745	GH43	ABF/BXL	Xylan
Pensub1|8222	GH5_22	EGL	Cellulose
Pensub1|5092	GH5_22	EGL	Cellulose
Pensub1|13043	GH10	XLN	Xylan
Pensub1|1444	GH11	XLN	Xylan
Pensub1|8824	CE5	AXE	Xylan
Pensub1|7375	GH11	XLN	Xylan
Pensub1|9764	GH3	BXL	Xylan
Pensub1|6031	GH43	ABF/BXL	Xylan
Pensub1|13445	GH10	XLN	Xylan
Pensub1|3512	GH93	ABX	Pectin
Pensub1|12441	GH10	XLN	Xylan
Pensub1|1664	GH51	ABF	Xylan/Pectin/Xyloglucan
Pensub1|5782	FAE	\	Pectin/Xylan
Pensub1|3511	GH3	BXL	Xylan
Pensub1|3364	GH54	ABF	Xylan/Pectin/Xyloglucan
Pensub1|12883	GH62	AXH	Xylan
Pensub1|12472	GH62	AXH	Xylan
Pensub1|6540	GH3	BGL/BXL	Cellulose/Xylan
Pensub1|14040	GH43	ABF/BXL	Xylan
Pensub1|6394	GH31	AXL	Xyloglucan
Pensub1|3476	GH27	AGL	Mannan
Pensub1|7184	CE1	AXE	Xylan
Pensub1|12473	GH43	ABF/BXL	Xylan
Pensub1|12887	GH43	ABF/BXL	Xylan
Pensub1|4845	GH43	ABF/BXL	Xylan
Pensub1|4850	GH54	ABF	Xylan/Pectin/Xyloglucan
Pensub1|3894	GH141	AFC	Xyloglucan
Pensub1|6724	GH54	ABF	Xylan/Pectin/Xyloglucan
Pensub1|3895	GH36	AGL	Mannan
Pensub1|12771	GH43	ABF/BXL	Xylan
Pensub1|3365	GH43	ABF/BXL	Xylan
Pensub1|6856	GH43	ABF/BXL	Xylan
Pensub1|12088	GH28	RGX	Pectin
Pensub1|12089	GH78	RHA	Pectin
Pensub1|12261	GH35	LAC	Pectin/Xyloglucan
Pensub1|6125	PL1_7	PLY	Pectin
Pensub1|13623	GH43	ABF/BXL	Xylan
Pensub1|4486	GH28	PGA	Pectin
Pensub1|3595	CE16	HAE	Xylan/Mannan/Xyloglucan
Pensub1|7919	GH67	AGU	Xylan
Pensub1|7918	GH30_7	EXL	Xylan
Pensub1|8192	GH28	RGX	Pectin
Pensub1|2516	GH35	LAC	Pectin/Xyloglucan
Pensub1|1378	GH88	UGH	Pectin
Pensub1|7796	AA9	LPMO	Cellulose
Pensub1|2020	FAE	\	Pectin/Xylan
Pensub1|1286	FAE	\	Pectin/Xylan
Pensub1|6610	GH1	BGL	Cellulose
Pensub1|1906	GH3	BGL	Cellulose
Pensub1|9846	CE5	AXE	Xylan
Pensub1|1352	CE16	HAE	Xylan/Mannan/Xyloglucan
Pensub1|13084	GH31	AGD	Starch
Pensub1|6750	CE1	AXE	Xylan
Pensub1|1735	PL1_4	PEL	Pectin
Pensub1|698	GH28	XGH	Pectin
Pensub1|10774	GH28	PGA	Pectin
Pensub1|699	CE8	PME	Pectin
Pensub1|2527	GH36	AGL	Mannan
Pensub1|8889	GH95	AFC	Xyloglucan
Pensub1|8310	CE12	RGAE	Pectin
Pensub1|6034	CE8	PME	Pectin
Pensub1|5036	GH5_5	EGL	Cellulose
Pensub1|6051	GH78	RHA	Pectin
Pensub1|520	CE12	RGAE	Pectin
Pensub1|13232	CE16	HAE	Xylan/Mannan/Xyloglucan
Pensub1|13583	GH78	RHA	Pectin
Pensub1|13618	GH28	RGX	Pectin
Pensub1|13338	GH78	RHA	Pectin

log2(FPKM+1)

0 5 10 15

P. subrubescens

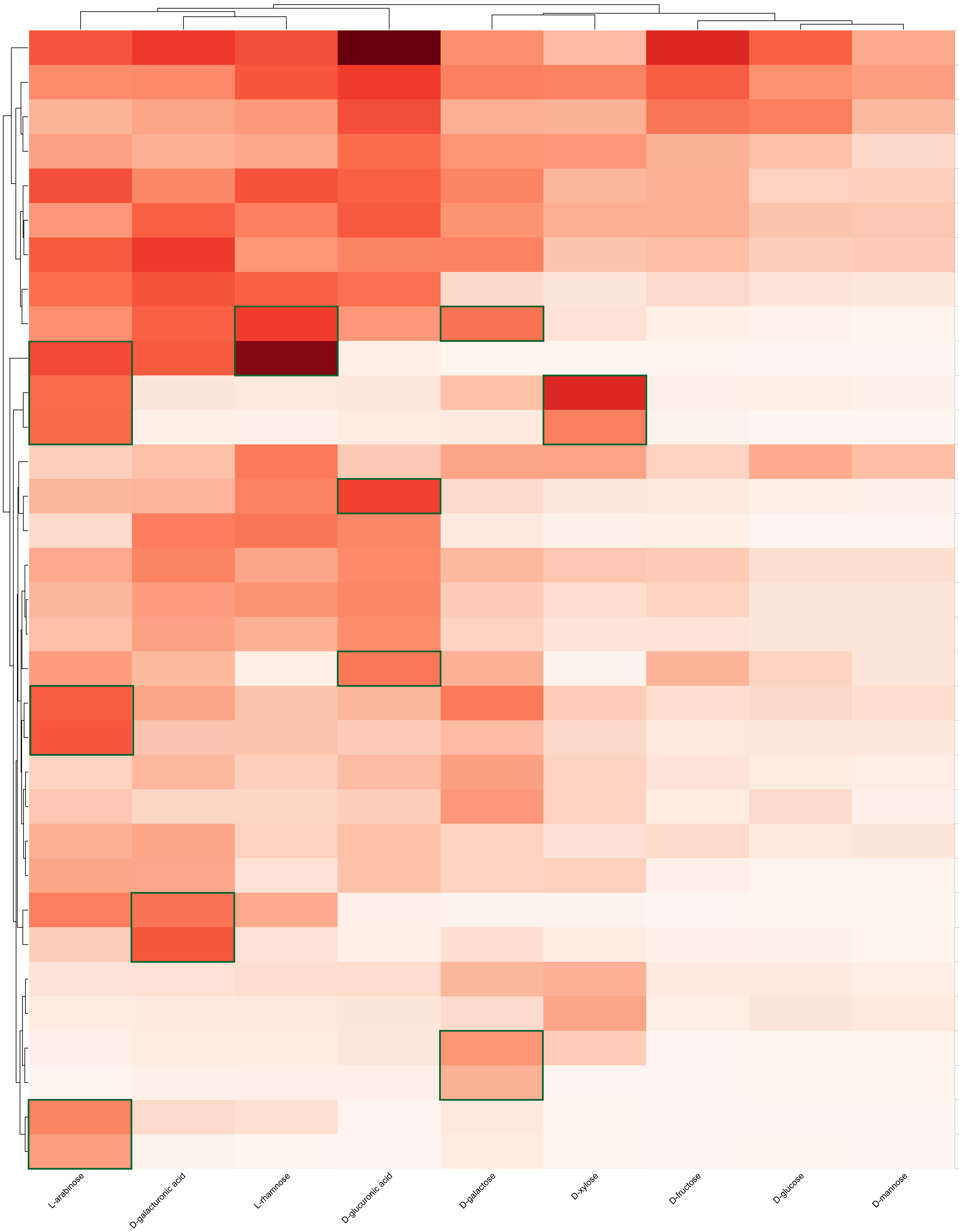

GeneID	CAZy family	Activity	Substrate
Trire_Chr|117523	GH3	BGL	Cellulose
Trire_Chr|116296	GH15	GLA	Starch
Trire_Chr|115357	GH31	AGD	Starch
Trire_Chr|113223	GH54	ABF	Xylan/Pectin/Xyloglucan
Trire_Chr|109040	GH3	BGL	Cellulose
Trire_Chr|110707	GH3	BGL	Cellulose
Trire_Chr|115491	GH36	AGL	Mannan
Trire_Chr|116633	GH28	RGX	Pectin
Trire_Chr|114300	GH27	AGL	Mannan
Trire_Chr|118807	GH105	URGH	Pectin
Trire_Chr|117721	GH30_7	EXL	Xylan
Trire_Chr|114049	GH3	BXL	Xylan
Trire_Chr|111151	CE1	AXE	Xylan
Trire_Chr|108972	CE5	AXE	Xylan
Trire_Chr|112598	GH2	MND	Mannan
Trire_Chr|109418	GH13_40	AGD	Starch
Trire_Chr|109861	GH3	BGL	Cellulose
Trire_Chr|114034	GH27	AGL	Mannan
Trire_Chr|113785	GH2	LAC	Pectin/Xyloglucan
Trire_Chr|114490	GH35	LAC	Pectin/Xyloglucan
Trire_Chr|114083	GH54	ABF	Xylan/Pectin/Xyloglucan
Trire_Chr|115441	GH27	AGL	Mannan
Trire_Chr|110029	GH1	BGL	Cellulose
Trire_Chr|113794	AA9	LPMO	Cellulose
Trire_Chr|111573	GH43	ABF/BXL	Xylan
Trire_Chr|115372	GH28	PGX	Pectin
Trire_Chr|117053	GH28	PGA	Pectin
Trire_Chr|114072	GH3	BGL	Cellulose
Trire_Chr|114117	CE1	AXE	Xylan
Trire_Chr|110436	GH95	AFC	Xyloglucan
Trire_Chr|113191	GH27	AGL	Mannan
Trire_Chr|110765	GH31	AXL	Xyloglucan
Trire_Chr|110735	GH62	AXH	Xylan

T. reesei

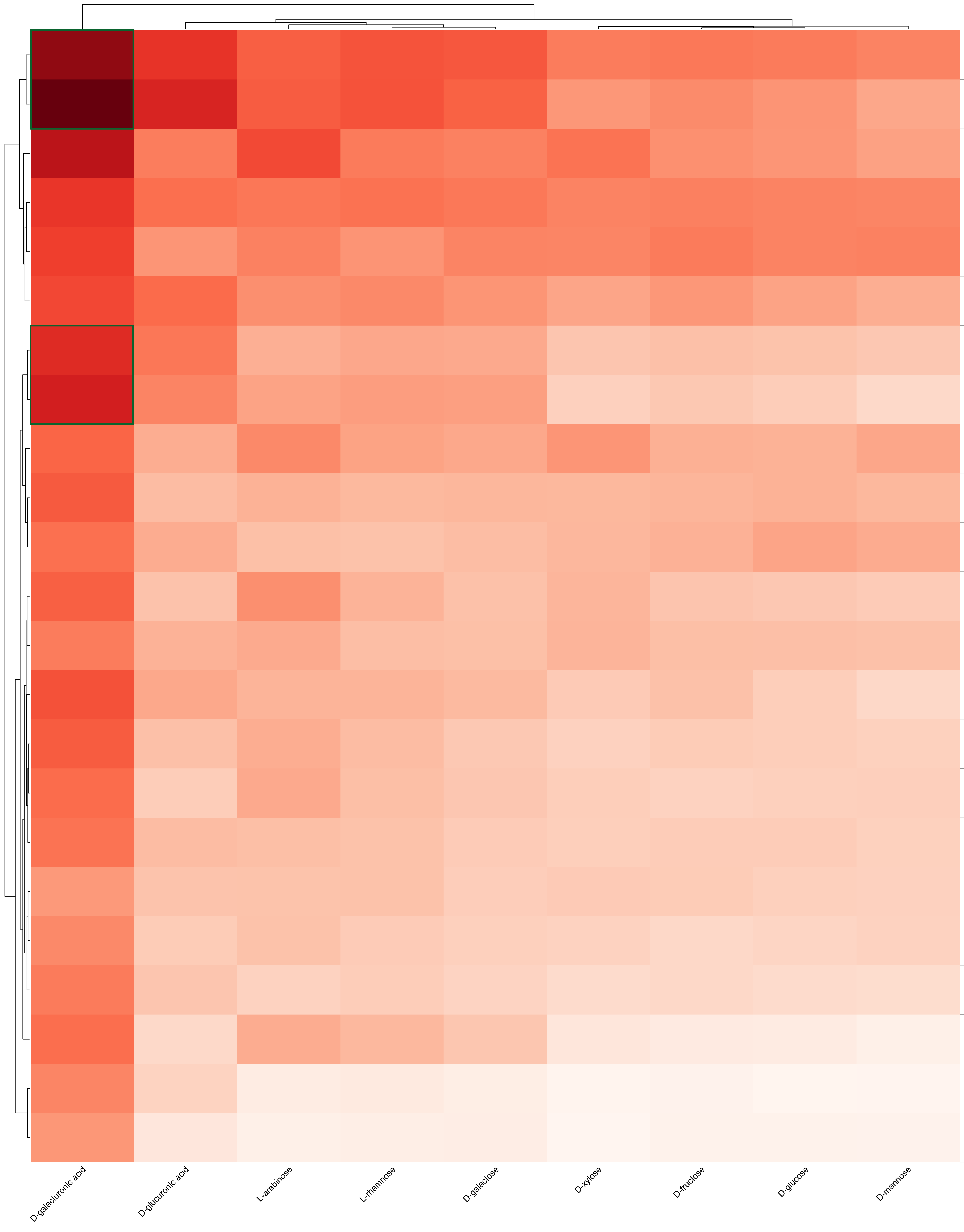

GeneID	CAZy family	Activity	Substrate
Phchr2|3027410	GH28	PGX	Pectin
Phchr2|2896433	GH43	ABF/BXL	Xylan
Phchr2|2989703	GH5_22	EGL	Cellulose
Phchr2|2990791	GH35	LAC	Pectin/Xyloglucan
Phchr2|2974470	GH3	BGL	Cellulose
Phchr2|3005667	GH27	AGL	Mannan
Phchr2|2989297	CE8	PME	Pectin
Phchr2|2989563	GH28	PGA	Pectin
Phchr2|2902324	GH1	BGL	Cellulose
Phchr2|2912243	CE15	GE	Xylan
Phchr2|2983017	AA9	LPMO	Cellulose
Phchr2|2908992	GH3	BGL	Cellulose
Phchr2|3030351	GH10	XLN	Xylan
Phchr2|2934294	CE8	PME	Pectin
Phchr2|3029839	GH5_7	MAN	Mannan
Phchr2|2910342	GH115	AGU	Xylan
Phchr2|3022485	GH31	AXL	Xyloglucan
Phchr2|1416762	GH12	XEG	Xyloglucan
Phchr2|3003144	GH3	BGL	Cellulose
Phchr2|2934334	GH78	RHA	Pectin
Phchr2|3042040	GH10	XLN	Xylan
Phchr2|2954077	GH51	ABF	Xylan/Pectin/Xyloglucan
Phchr2|2979830	GH28	RGX	Pectin

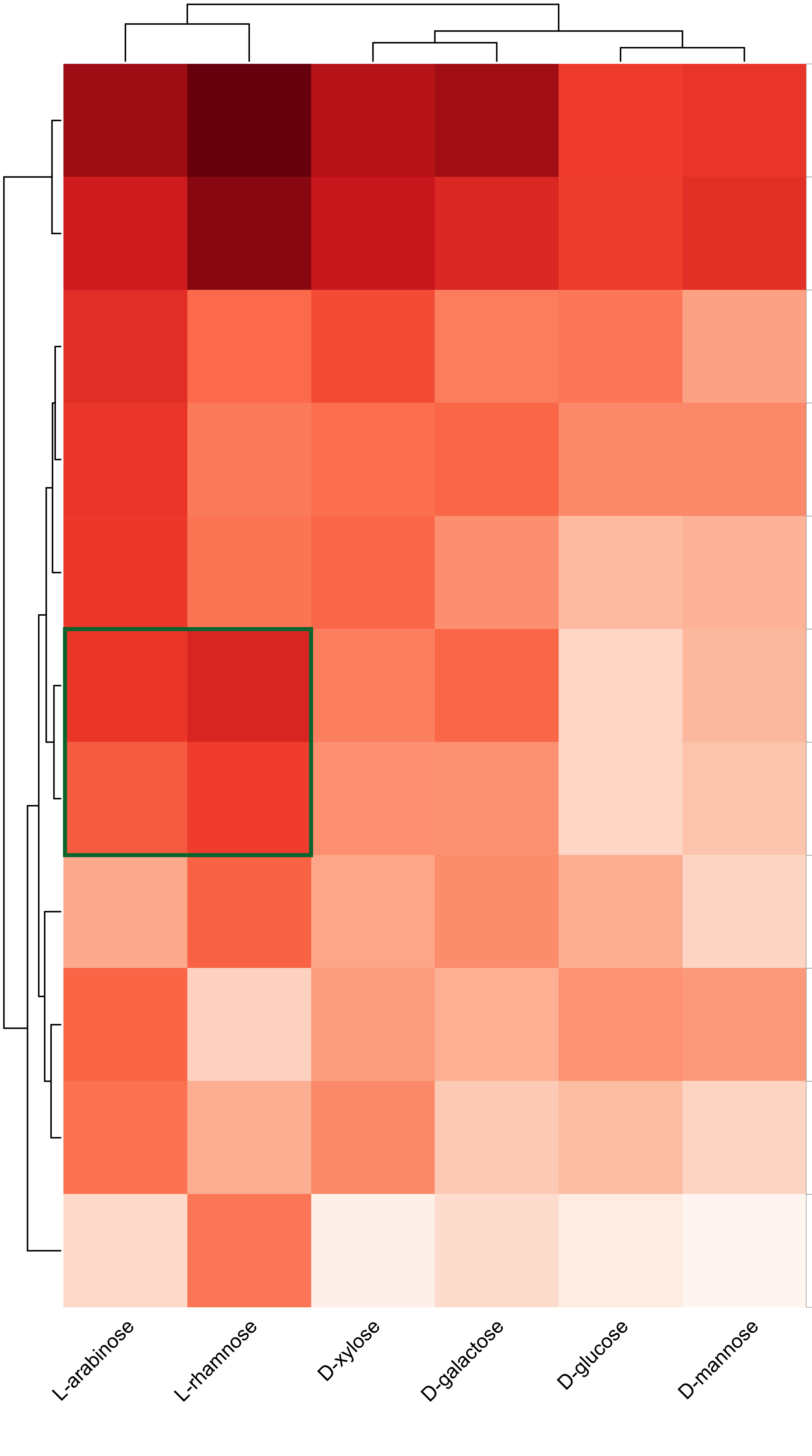

GeneID	CAZy family	Activity	Substrate
Dicsqu464_1|1040936	GH35	LAC	Pectin/Xyloglucan
Dicsqu464_1|882755	GH45	EGL	Cellulose
Dicsqu464_1|465156	AA9	LPMO	Cellulose
Dicsqu464_1|825072	GH5_7	MAN	Mannan
Dicsqu464_1|918783	GH7	CBH	Cellulose
Dicsqu464_1|513778	GH10	XLN	Xylan
Dicsqu464_1|933193	CE15	GE	Xylan
Dicsqu464_1|841467	GH28	PGA	Pectin
Dicsqu464_1|946952	GH7	CBH	Cellulose
Dicsqu464_1|945667	GH10	XLN	Xylan
Dicsqu464_1|933288	GH10	XLN	Xylan
