# Supplementary figures and images for: Comparative Genomics and Transcriptomics Analyses Reveal Divergent Plant Biomass-Degrading Strategies in Fungi
Source: J Fungi (Basel). 2023 Aug 18;9(8):860. doi: 10.3390/jof9080860 (PMC10455118; doi:10.3390/jof9080860)

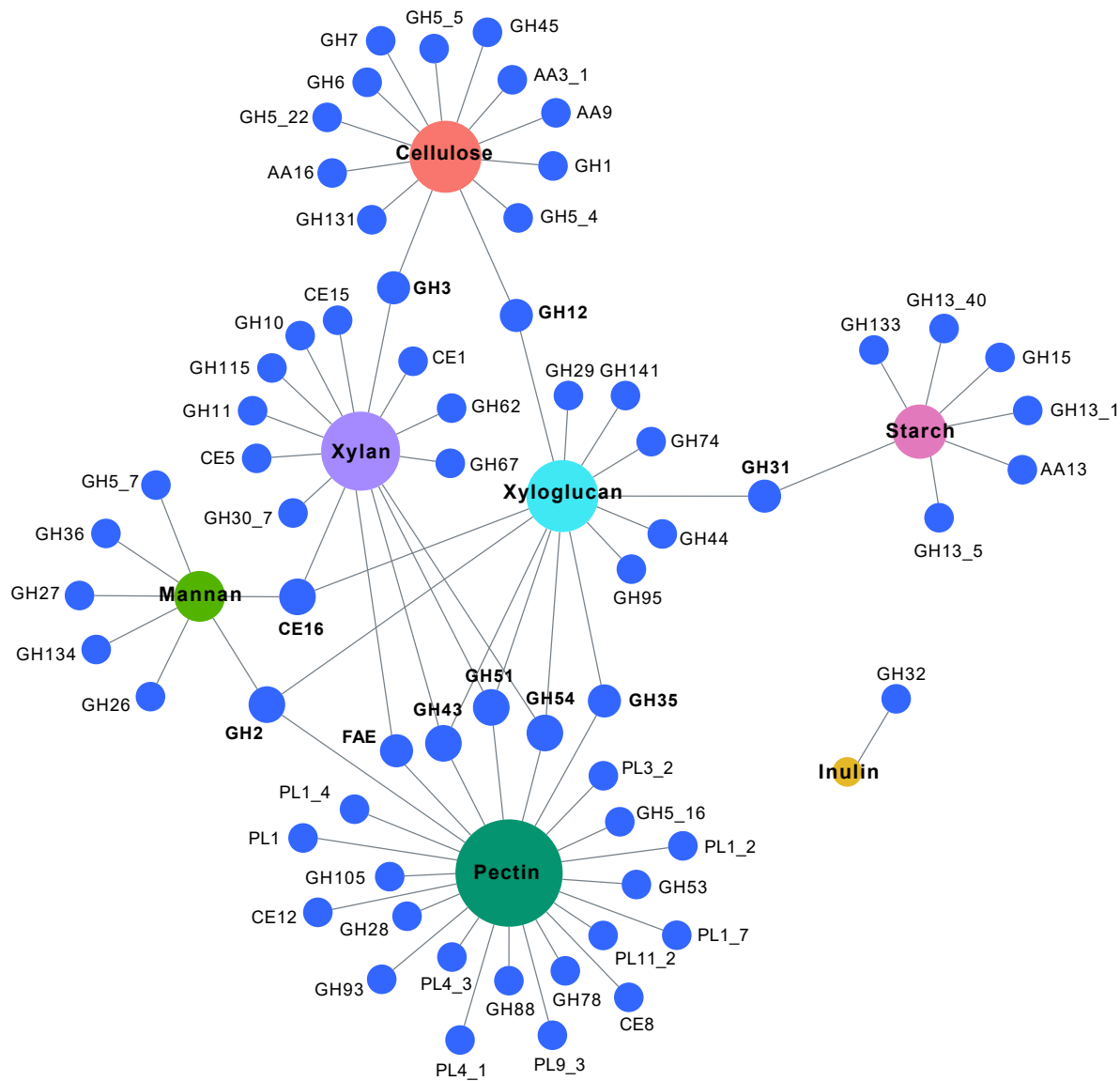

Supplement: Supplementary file 1 [file jof-09-00860-s001.zip › Figure S1.pdf]

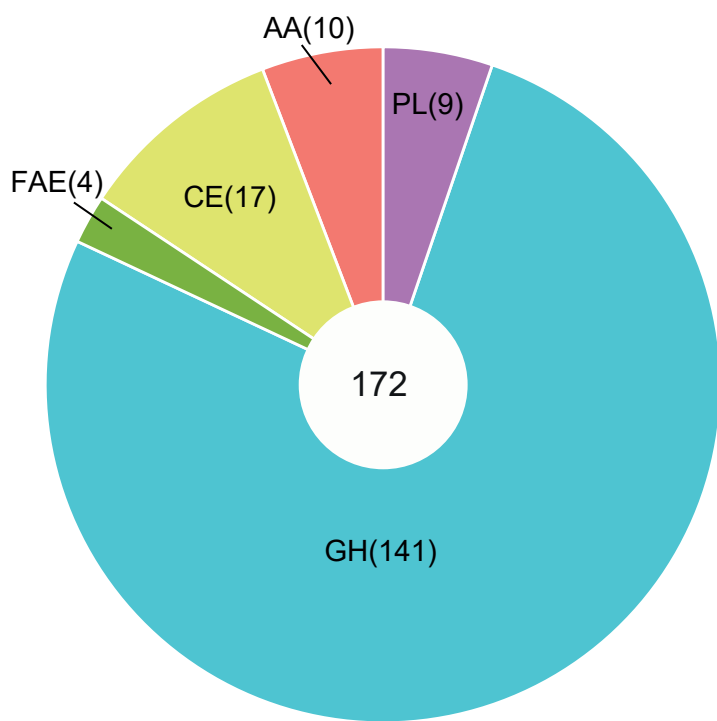

A. *A. niger*

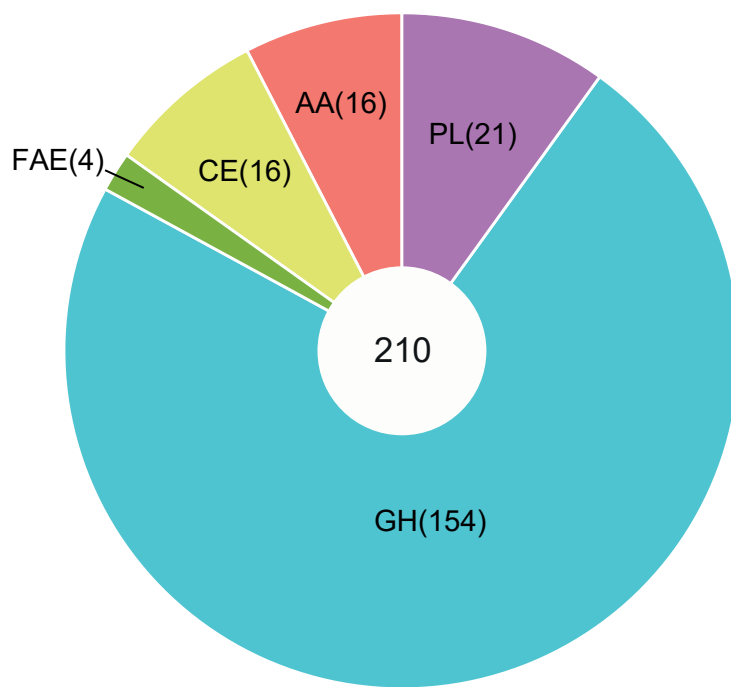

B. *A. nidulans*

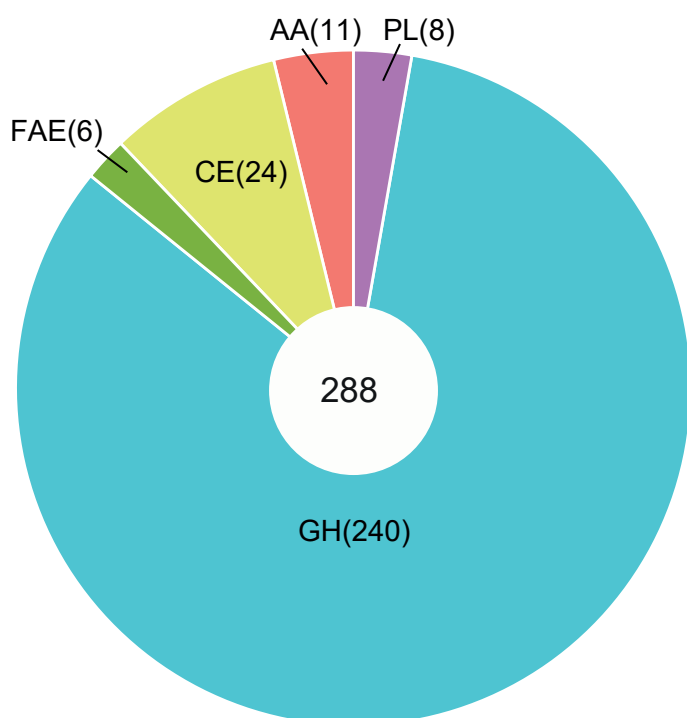

C. *P. subrubescens*

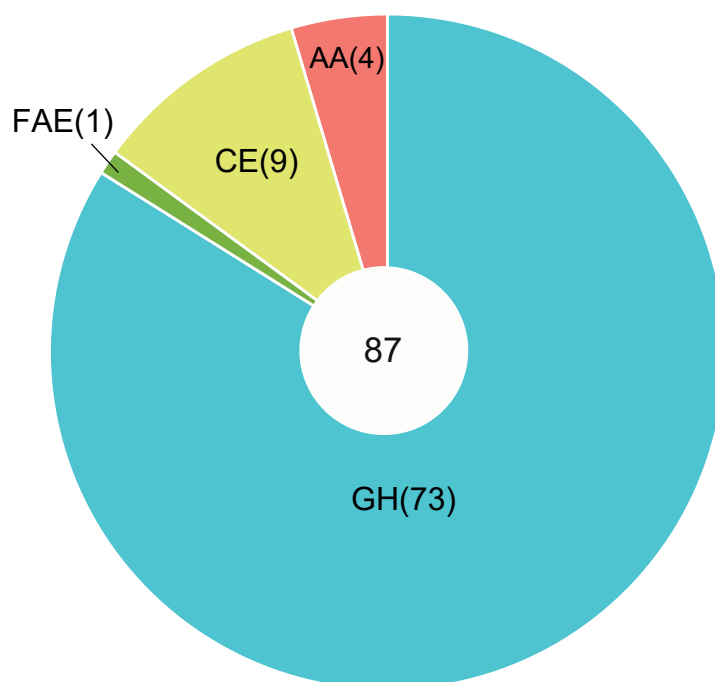

D. *T. reesei*

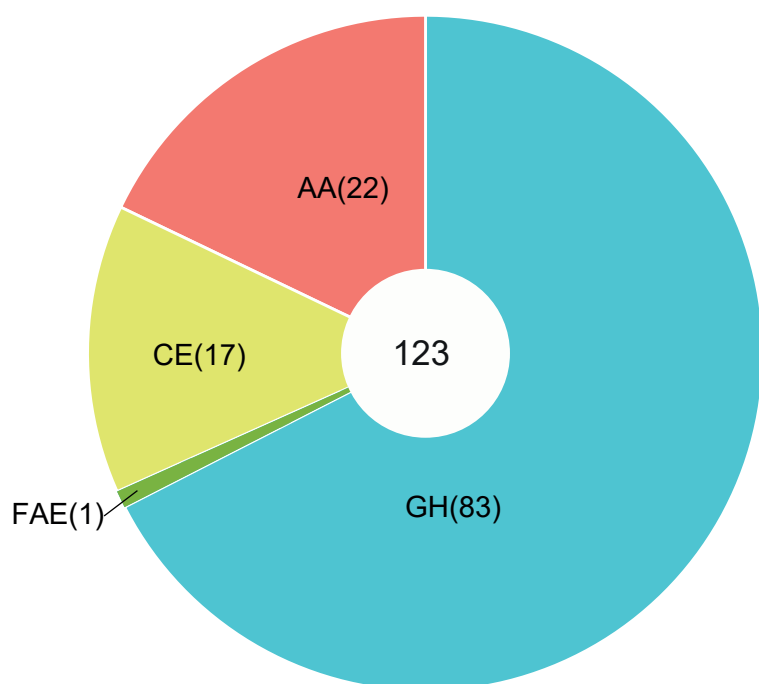

E. *P. chrysosporium*

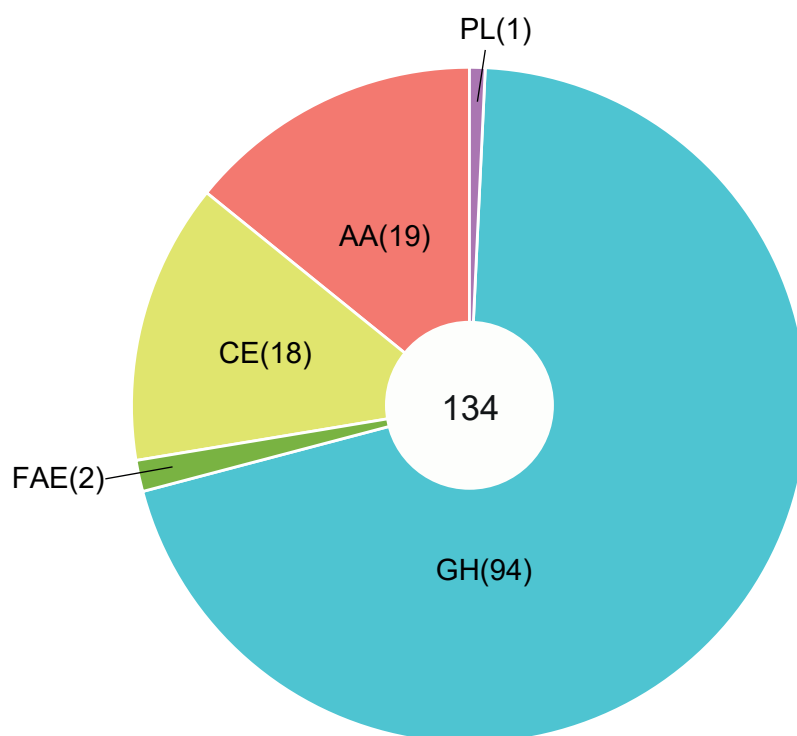

F. *D. squalens*

Supplement: Supplementary file 1 [file jof-09-00860-s001.zip › Figure S2.pdf]
